# The Effect and Implication of Social Media Platforms on Plastic Cosmetic Surgery: A Cross-sectional Study in Saudi Arabia From 2021 to 2022

**DOI:** 10.1093/asjof/ojad002

**Published:** 2023-01-18

**Authors:** Ahmed AlBahlal, Norah Alosaimi, Manar Bawadood, Abdulrahman AlHarbi, Fatema AlSubhi

## Abstract

**Background:**

Aesthetic procedures are considered one of the most commonly performed procedures in the medical field. Social media (SM) reflects the electronic platforms that deliver an enormous amount of information to different users and enable them to share their content and experience with others at the simple click of a button. In our modern era, SM platforms affect different angles of our lives, from a simple detail to a significant complex aspect.

**Objectives:**

To evaluate the effect of different SM platforms on plastic cosmetic surgery in Saudi Arabia.

**Methods:**

The authors conducted a cross-sectional study in 2021, employing a random sampling technique with a sample size of 2249 participants (ages 12 to >50). They included all plastic cosmetic interventions, and excluded reconstructive and traumatic interventions.

**Results:**

It was reported that 56.7% were not interested in doing surgical or non-surgical cosmetic interventions, while 43.3% were interested. Those influenced by SM platforms were either interested or not interested in doing cosmetic interventions. Snapchat (Santa Monica, CA) was the most commonly influential SM platform. In addition, 35.9% answered that surgeons’ advertisements affected their decision to seek plastic surgery consultations. Photograph editing applications made 46% of participants look better and more confident to post and share their pictures.

**Conclusions:**

Our analysis showed that those influenced by SM platforms to seek cosmetic treatment were comparatively more interested in cosmetic treatment, with Snapchat being the most influential platform. Therefore, further studies to evaluate the impact of SM platforms among plastic surgeons are encouraged.

**Level of Evidence: 4:**

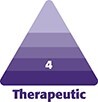

There are many elective procedures in the scope of the medical field, with a considerable portion occupied by aesthetic procedures. Multiple factors play a role in increasing the popularity of these procedures, such as body image dissatisfaction, looking for perfection, a surgeon's reputation, and social media (SM), which connects the dots between them. SM terms reflect a diversity of platforms that target different users. Historically, surgeons used their private web pages. Nowadays, modern surgeons are using SM platforms instead.^1^ In addition, knowledge is a known prerequisite for the change in health behavior. To our knowledge, there is meager information regarding the effect and implication of SM platforms on plastic cosmetic procedures in Saudi Arabia.

The impact of SM can be beneficial, but is not well-understood by people on SM as to how it reflects cosmetic surgery.^2^ Multiple social platforms such as Snapchat (Santa Monica, CA) and Instagram (Menlo Park, CA) have editing features like filters and variants of applications that alter facial characteristics such as softening wrinkles as well as changing eyes and nose sizes before posting photos in public. The increase in the use of filtered selfies led cosmetic surgeons to coin a new term—Snapchat dysmorphia—which describes the desire of patients for their cosmetic surgery results to look like filtered photos of themselves.^3^ Instagram was the most influential application, followed by Snapchat, and then Twitter (San Francisco, CA); 50% of the participants routinely applied Snapchat filters, and 42% decided to undergo facial changes after using Snapchat filters.^1^ TikTok (Culver City, CA) is an imaginative way to show plastic surgery video content and has more prominent total engagement than Instagram.^4^ A study conducted in Saudi Arabia in 2018 showed that most of their participants said yes to the effect of before-and-after pictures on SM and agreed on the influence of the surgeon's self-advertisement on the trend of cosmetic surgery.^5^ A study was held in different regions of Saudi Arabia among 911 participants of males and females to evaluate the effect of SM on the Saudi population to undergo a cosmetic procedure to correlate the impact of SM with socio-demographic factors; the results showed that the majority were influenced by before-and-after comparison pictures, the desire to appear good in photographs, and the advertisement by surgeons and different television cosmetic shows.^6^ The strong attachment to SM platforms makes it an advertisement area for aesthetic surgeries. As SM has become unavoidable in our modern society, different specialties have taken their seats on various platforms; because of that, asking questions and answering queries has become more feasible and accessible. With the power of SM, physicians first must comprehend how to use these sites and how different platforms relate to one another.^7^ The main objective of this study was to evaluate the effect of different SM platforms on plastic cosmetic surgery in Saudi Arabia. We hypothesize that there is an effect of SM platforms on the rate of plastic cosmetic procedures.

A study was conducted on 100 patients of cosmetic clinics by filling out a survey regarding the aesthetic procedure posts that attract them on SM (Appendix). It showed a discrepancy between the content that could engage plastic surgeons vs the content that could engage patients. When we can better comprehend the patients’ perspectives, we will bridge the gap and create a digital presence that is more tailored to the patients’ desires.^8^ An article selected and analyzed about 26 studies that discuss the effect of what is displayed in SM regarding cosmetic promotion and sponsorship, and found that there is a clear, useful impact of SM in the plastic field despite general ethical guidelines, but promotion companies’ commitment toward them was unclear. The article also recommended monitoring this by using health care providers to create a standardized program.^2^ A systematic review article reviewed 28 articles (2010-2017) discussing ethical principles and concepts of SM use in different surgical subspecialties, including 6 research in the plastic surgery field. Despite the ability of SM in education, sharing experiences, discussing opinions with many experts in the same field, and keeping up with development, it is an environment that allows many offenses to happen, so it is recommended that plastic surgery experts pay special care to professionalism when using SM.^9^ A study was performed in a plastic surgery clinic located in Hangzhou, China during the COVID-19 pandemic to report how SM has helped patients to reach plastic surgeons, resulting in the increase of the clinic's revenue by 22.8% compared with the same period last year. There was an upward trend toward online consultations and the cost was spent on time. However, staff resources declined by 20%.^10^ A cross-sectional study was conducted using an electronic survey of 399 patients in plastic surgery clinics at King Abdulaziz University Hospital in Riyadh. This study has indicated that SM is not responsible for normalizing the culture of perfection and optimal beauty. But that also depends on television programs and advertisements, as they also have a role in convincing people to undergo plastic surgery and giving hope to patients who are unsatisfied with their looks.^5^ Another study assessed the influence of SM on the volume of aesthetic cases performed through a built-up chief resident clinic and its utility in patient enlistment. Chief residents increased the number of aesthetic surgery cases they performed after residents included their clinic on an online SM website. This impact enhances the effect SM and web apprentices have on plastic surgery training.^11^

Plastic surgery posts on Instagram and Twitter to assess platform substance contrast quantitatively and subjectively. An add-up of 3867 Twitter posts and 5098 Instagram posts were included in this experiment. The day-by-day add-up to post volume for the 1-month term was higher on Instagram than on Twitter. In general, post-engagement was particularly higher on Instagram compared with Twitter. Plastic specialists and plastic surgery clinics have spoken to the larger part of accounts by posting on both stages with #PlasticSurgery. Recognizable patient features are more common on Instagram. Most of the posts on Instagram are promotional, results-based, or unrelated to plastic surgery. On the other hand, Tweets were mostly educational.^7^ SM has increased the penetration of cosmetic surgery into the public's consciousness. Plastic surgeons are keenly interested in performing desired surgery and being reimbursed for doing so, but they must strive to advertise professionally and ethically. The American Society of Plastic Surgeons monitors this ever-evolving landscape through its Social Media Subcommittee. It will continue to provide education and guidance to its members and the public.^12^ A study analyzed the usefulness of SM and the internet in interacting with patients during their first consultation in public and private plastic surgery clinics between September 2017 and December 2017, by a series of 18-question surveys with 313 filled questionnaires: 200 in the aesthetic group (AG) and 113 in the public group (PG). The AG of patients focused on the before-and-after photographs and the surgeon's credentials. The PG patients saw that SM was the worst source of information, but the AG patients appreciated SM as a good source of information.^13^ The current generation in plastic surgery is called the millennial generation. While studying the differences between the millennials and the generations before them, it has become clear that their characteristics are uniform.^14^ A study identified trends in using online reviews among plastic surgeons who performed gender confirming surgeries in the United States and assessed the influence of SM and other factors on online reviews that were obtained from 3 of the most popular physician-rating websites (HealthGrades.com [Denver, CO], Vitals.com [Lyndhurst, NJ], and WebMD.com [New York, NY]). Online ratings and comments were compared with age, years of practice, academic appointment, type of practice, geographic region of practice, SM use, and indexed articles. Seventy-five plastic surgeons were included; a total of 69 plastic surgeons (92.9%) had at least 1 SM account with at least 1 follower. The most popular SM account was LinkedIn (Sunnyvale, CA) (82.7%), followed by Facebook (Menlo Park, CA) accounts (65.3%). The study did not find a significant association between online ratings and SM accounts. Nevertheless, the study found that the number of positive comments was significantly higher among plastic surgeons with YouTube (San Bruno, CA), Instagram, or Facebook pages, which may add evidence to the importance of SM on online reviews at various levels.^15^ A cross-sectional study was conducted in Riyadh, Saudi Arabia, between September 2017 and October 2018, with a sample size of 1449 females visiting the facial plastic clinic at King Abdulaziz Medical City, aimed to assess the impact of SM on aesthetic procedures which had influenced 68% of participants.^1^ A study was held in different regions of Saudi Arabia among 911 participants of males and females to evaluate the effect of SM on the Saudi population to undergo a cosmetic procedure to correlate the impact of SM with socio-demographic factors; the results showed that the majority of participants were influenced by before-and-after comparison pictures, the desire to appear good in photographs, and the advertisement by surgeons and different television cosmetic shows.^6^ A cross-sectional study was conducted in Riyadh among 816 female university students to determine the effects of SM on their decision to undergo cosmetic treatments. The results showed that most participants interested in cosmetic procedures were influenced by advertisements, celebrities, and SM influencers. In addition, they found that the experiences of their family members or friends who had undergone cosmetic treatments were contributing factors that increased the desire to try out these procedures.^16^ A study analyzed the impact of SM and photo editing on cosmetic surgery attitudes. It used a survey administered through online platforms to 252 participants from July 1 to September 19, 2018. The study's findings suggest that using SM and photo editing apps may increase the acceptance of cosmetics.^3^

## METHODS

This cross-sectional study was conducted in Saudi Arabia with 2248 participants. The study included participants aged 12 and above, and excluded participants less than 12 years old, patients who underwent reconstructive interventions, and traumatic patients. The data were collected through an online questionnaire by Google (Mountain View, CA) form, not showing any nominative information. The questionnaire was electronically distributed in all regions of Saudi Arabia. After verification, data were transferred to the statistical database directly. Participant consent was implied by completing the questionnaire and reading the aims and goals of the study. It was provided in Arabic and English versions. The survey was administered through online platforms from August 11 to September 29, 2021. Data were entered using Microsoft Excel 365 (Redmond, WA), and all statistical analyses were conducted using SPSS 25 v (IBM, Armonk, NY). We used a *t*-test. Statistical significance was set at *P* < .05. Numeric data are presented as mean (+ or −) SD if normally distributed, and by median and interquartile range if not normally distributed; categorical data are presented as a percentage and are compared using the chi-squared test. We obtained ethical approval for the study, according to the declaration of Helsinki from the Biomedical Ethics Research Committee of Prince Sultan Military Medical City, Riyadh, Saudi Arabia.

## RESULTS

**Figure 1.**
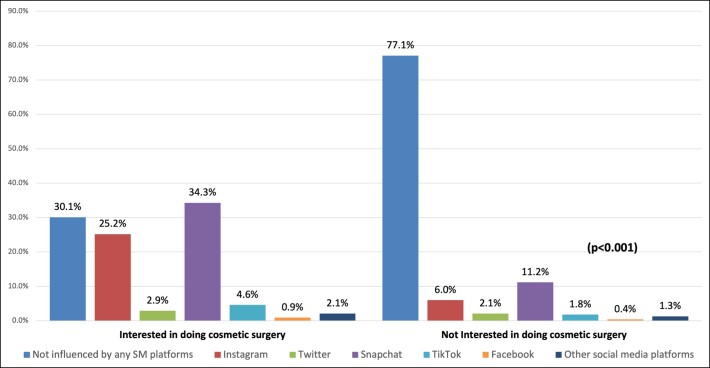
Type of social media that influenced the participants to consider cosmetic interventions.

**Figure 2.**
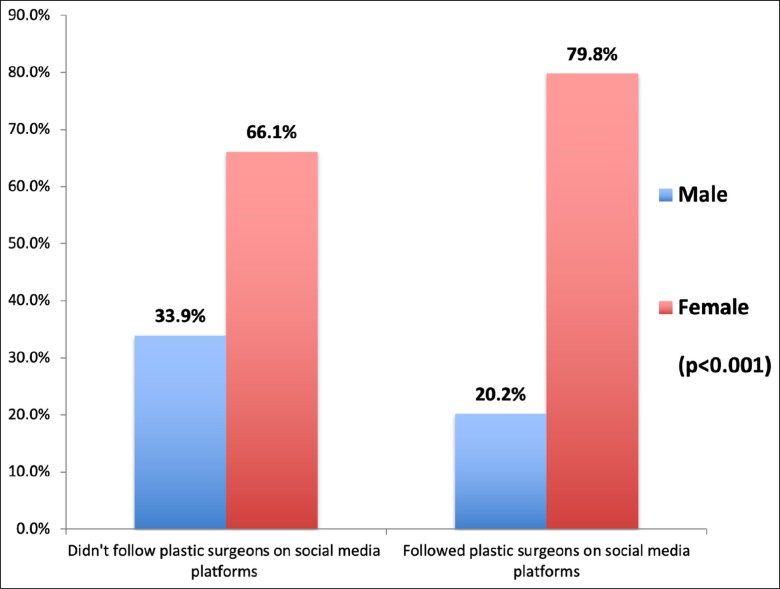
Following plastic surgeons on social media platforms and its relationship with gender.

**Figure 3.**
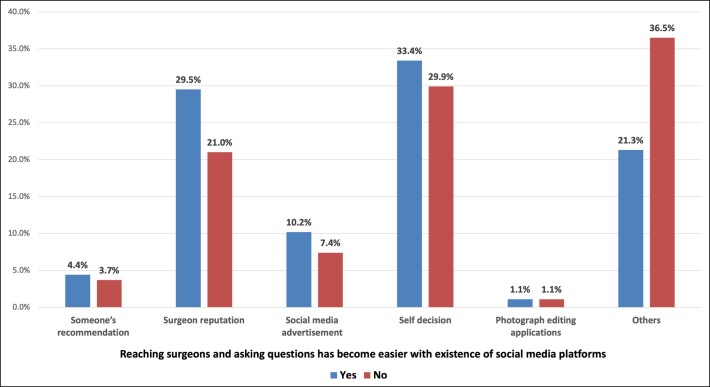
Reasons to accept for visiting plastic surgery consultation.

**Figure 4.**
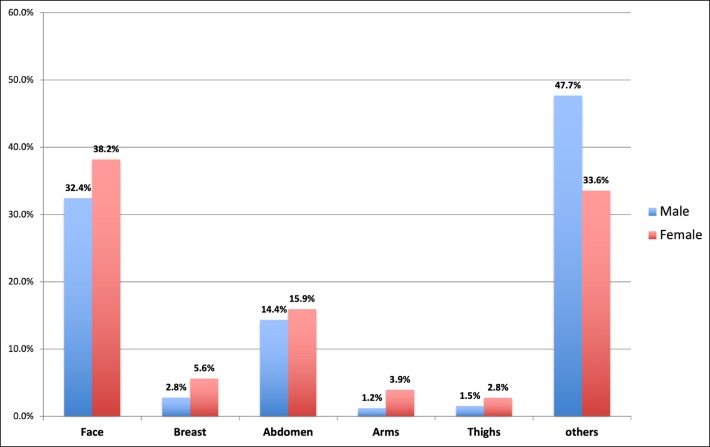
Area of interest for cosmetic interventions based on gender (*P* < .001).

**Figure 5.**
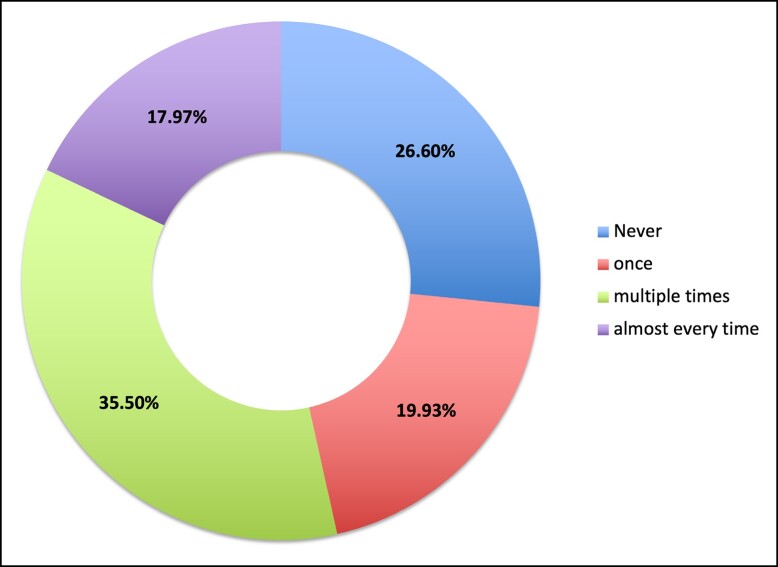
The frequency of using applications that have photograph editing abilities and filters and applying them.

**Figure 6.**
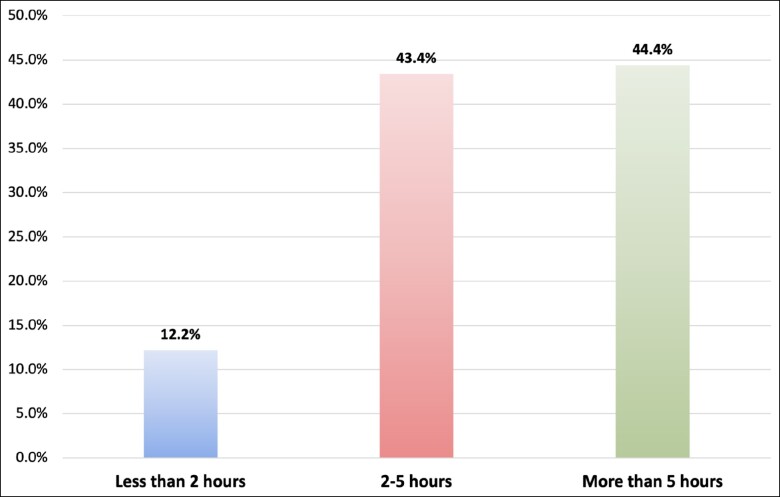
How often do participants use social media per day?

**Figure 7.**
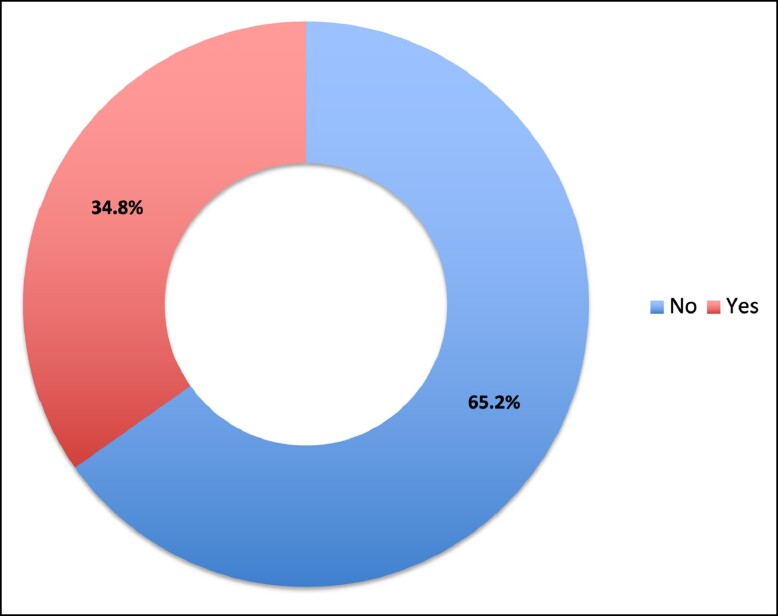
Did photograph editing applications affect participants’ decisions in seeking plastic surgery consultations and interventions?

**Figure 8.**
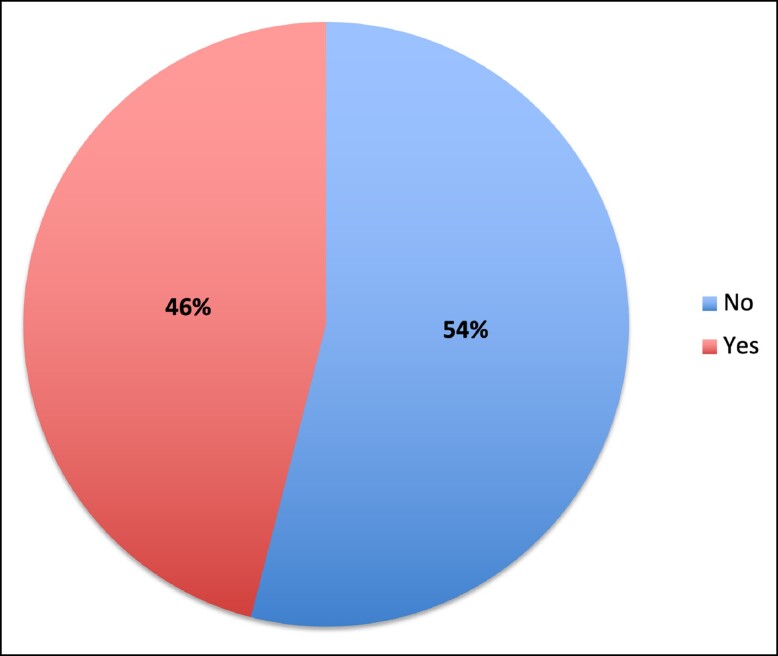
Did photograph editing applications make participants look better and more confident to post or share on different social media platforms?

Our analysis included responses from 2248 participants. The socio-demographic details showed that 46.2% of them belonged 21 to 30 year-old group, 71.2% were females, 91.4% were Saudi citizens, 59.1% belonged to the Western region of the Kingdom of Saudi Arabia, 51.7% were single, 80.8% had University level education, 40.7% were employed, and 29% had no salary. It was found that 43.3% (*n* = 973) were interested in doing cosmetic interventions, where the majority of the participants belonged to the 21 to 30 year-old group compared with the other age groups (*P* < .001). Female and single participants were significantly more interested in cosmetic treatment than their counterparts (*P* < .001). Participants who did not have salaries were not interested in doing such treatment compared with others who had a salary, which showed a statistically significant difference (*P* = .022) (Table 1). Participants who reported that SM influenced them to seek cosmetic treatment were comparatively more interested in doing cosmetic treatment, which showed a statistically significant relationship (*P* < .001). It was reported that 56.7% (*n* = 1275) were not interested in doing surgical or non-surgical cosmetic interventions, while 43.3% were interested. Those who were influenced by SM platforms were either interested or not interested in doing cosmetic interventions. Snapchat was the most influential SM platform (34.3%), followed by Instagram (25.2%) among those interested. For those who were not interested in cosmetic interventions, Snapchat was the most influential (11.2%), followed by Instagram (6.0%) (Figure 1). It was found that only 6% (*n* = 135) and 15.2% (*n* = 342) underwent surgical cosmetic and non-surgical treatments, respectively. The association observed between SM influence and cosmetic treatment that was done where participants who used some type of SM such as Snapchat, Instagram, Twitter, TikTok, and Facebook underwent surgical and non-surgical cosmetic treatments was significantly higher than that for people who did not use any SM platforms (*P* < .001). It was reported by 35.9% of the participants that surgeons’ advertisements affected the decision in seeking plastic surgery consultations and treatment, which was higher in participants who reported that SM platforms influenced them to seek cosmetic treatment than in those who reported that SM did not influence them (*P* < .001) (Table 2). About 60.3% (*n* = 1355) reported that their salary affected the decision to undergo plastic cosmetic interventions, but this did not show any statistically significant association with their reported salary (*P* = .502) (Table 3). Female participants followed plastic surgeons on SM platforms more than males (*P* < .001) (Figure 2). It was reported by 87.9% (*n* = 1977) that reaching surgeons and asking questions has become easier with the existence of SM, and this was most influenced by the surgeon's reputation and SM advertisement (*P* = .021) (Figure 3). Participants were mostly interested in cosmetic intervention on their face (36.5%), followed by the abdomen (15.3%), and females were comparatively more interested than males (*P* < .001) (Figure 4). About 35.0% (*n* = 788) of the participants reported that before-and-after pictures affected their decision to seek plastic surgery consultations and interventions, and for the surgeons who posted these photos, the advertisement was an influential factor (59.6%) (*P* < .001). Also, this was more reported among the age groups of 21 to 30 years, 31 to 40 years, and 31 to 40 years than the other age groups (*P* = .005) (Table 4). It was reported by 34.83% (*n* = 783) that photograph-editing applications affected their decision in seeking plastic surgery consultations and interventions, which showed a statistically significant association with surgeon advertisements (*P* < .001) (Table 5). It was found in 41.2% (*n* = 926) that the desire to appear better in photos motivated them to undergo a cosmetic intervention, and this did not show any significant relationship with participants’ educational level (*P* = .872) (Table 6). The most commonly used SM, in general, among the participants was Snapchat (38%), followed by Twitter (20.2%), Instagram (18.8%), and TikTok (10.8%) (Table 7). Results showed that, of those participants who used applications with photograph-editing abilities and filters, 35.5% used these options multiple times per day, whereas 26.6% never used them, 19.33% used them at least once per day, and 17.97% used them all the time (Figure 5). The analysis of time spent on SM showed that about 44.4% used it for more than 5 hours, 43.4% used it for 2 to 5 hours per day, and 12.2% used it for less than 2 hours per day (Figure 6). About 46% think that the photo editing application made them look better and more confident to post/share their photos on different SM platforms (Figure 7). It was reported by 34.8% that photograph-editing applications affected their decision in seeking plastic surgery consultation and interventions (Figure 8), and about 46% think that the photograph-editing applications made them look better and more confident to post/share their photos on different SM platforms (Figure 7). The content most searched for was “before-and-after photographs” of cosmetic treatment (30.29%), followed by information about procedures (18.28%). There was a statistically significant association observed between the frequency of SM usage and “wanted to see” content, where participants who used SM multiple times per day used it to see “before-and-after photographs” (33.08%) compared with other purposes (*P* < .001) (Table 8).

**Table 1.** Relationship Between Interest in Doing Cosmetic Interventions and Socio-demographic Characteristics

Demographic			Interest in doing cosmetic Rx		Total	*P* value
			No	Yes		
Age (years)	12-20	N	173	107	280	<.001
		%	13.6%	11.0%	12.5%	
	21-30	N	559	480	1039	
		%	43.9%	49.3%	46.2%	
	31-40	N	214	195	409	
		%	16.8%	20.0%	18.2%	
	41-50	N	220	140	360	
		%	17.3%	14.4%	16.0%	
	> 51	N	108	52	160	
		%	8.5%	5.3%	7.1%	
Gender	Male	N	452	196	648	<.001
		%	35.5%	20.1%	28.8%	
	Female	N	822	778	1600	
		%	64.5%	79.9%	71.2%	
Nationality	Saudi	N	1154	900	2054	.127
		%	90.6%	92.4%	91.4%	
	Non-Saudi	N	120	74	194	
		%	9.4%	7.6%	8.6%	
Regions	North	N	213	134	347	.080
		%	16.7%	13.8%	15.4%	
	East	N	86	58	144	
		%	6.8%	6.0%	6.4%	
	West	N	754	574	1328	
		%	59.2%	58.9%	59.1%	
	South	N	49	47	96	
		%	3.8%	4.8%	4.3%	
	Center	N	172	161	333	
		%	13.5%	16.5%	14.8%	
Marital status	Single	N	641	522	1163	<.001
		%	50.3%	53.6%	51.7%	
	Married	N	584	380	964	
		%	45.8%	39.0%	42.9%	
	Divorced	N	33	64	97	
		%	2.6%	6.6%	4.3%	
	Widow	N	16	8	24	
		%	1.3%	0.8%	1.1%	
Education level	No education	N	4	2	6	.090
		%	0.3%	0.1%	0.2%	
	School (Primary,Secondary, high)	N	261	165	426	
		%	20.5%	16.9%	19.0%	
	University (Bachelor, Master, PhD)	N	1009	807	1816	
		%	79.2%	82.9%	80.8%	
Employment status	Unemployed	N	271	210	481	.079
		%	21.3%	21.6%	21.4%	
	Student	N	443	312	755	
		%	34.8%	32.0%	33.6%	
	Self-employed	N	43	53	96	
		%	3.4%	5.4%	4.3%	
	Employed	N	517	399	916	
		%	40.6%	41.0%	40.7%	
Monthly income	No salary	N	384	267	651	.022
		%	30.1%	27.4%	29.0%	
	<5000 SR	N	321	252	573	
		%	25.2%	25.9%	25.5%	
	5000-10.000 SR	N	226	212	438	
		%	17.7%	21.8%	19.5%	
	11.000-20.000 SR	N	263	192	455	
		%	20.6%	19.7%	20.2%	
	21.000-40.000	N	69	35	104	
		%	5.4%	3.6%	4.6%	
	>40.000 SR	N	11	16	27	
		%	0.9%	1.6%	1.2%	

SR, Saudi Riyal.

**Table 2.** Type of Social Media Influence and Its Relationship With Practice and Attitude Toward Cosmetic Interventions

Patient experience		Social media influence on cosmetic interventions							Total	*P* value
		Not influenced by any SM platforms	Instagram (Menlo Park, CA)	Twitter (San Francisco, CA)	Snapchat (Santa Monica, CA)	TikTok (Culver City, CA)	Facebook (Menlo Park, CA)	Other SM apps		
Done surgical cosmetic interventions	No	1231	292	49	435	62	11	33	2113	<.001
		58.3%	13.8%	2.3%	20.6%	2.9%	0.5%	1.6%	94.0%	
	Yes	44	30	6	42	6	3	4	135	
		32.6%	22.2%	4.4%	31.1%	4.4%	2.2%	3.0%	6.0%	
Done non-surgical cosmetic interventions	No	1183	245	46	339	51	10	32	1906	<.001
		62.1%	12.9%	2.4%	17.8%	2.7%	0.5%	1.7%	84.8%	
	Yes	92	77	9	138	17	4	5	342	
		26.9%	22.5%	2.6%	40.4%	5.0%	1.2%	1.5%	15.2%	
Surgeon advertisement affected the decision in seeking plastic surgery consultations and interventions	No	1021	143	23	196	29	7	21	1440	<.001
		70.9%	9.9%	1.6%	13.6%	2.0%	0.5%	1.5%	64.1%	
	Yes	254	179	32	281	39	7	16	808	
		31.4%	22.2%	4.0%	34.8%	4.8%	0.9%	2.0%	35.9%	

SM, social media.

**Table 3.** Relationship Between Salary Affecting the Decision to Undergo Plastic Cosmetic Interventions and Monthly Income

Monthly income (SR)		Salary affected the decision to undergo plastic cosmetic interventions		Total	*P* value
		No	Yes		
Don’t have income	N	278	373	651	.502
	%	31.1%	27.5%	29.0%	
<5000	N	217	356	573	
	%	24.3%	26.3%	25.5%	
5000-10,000	N	172	266	438	
	%	19.3%	19.6%	19.5%	
11,000-20,000	N	172	283	455	
	%	19.3%	20.9%	20.2%	
21.000-40,000	N	44	60	104	
	%	4.9%	4.4%	4.6%	
>40,000	N	10	17	27	
	%	1.1%	1.3%	1.2%	

SR, Saudi Riyal.

**Table 4.** Effect of Pictures on Decision-Making to Seek Plastic Surgery and its Relationship With Other Characteristics

Patient demographic			Before and after pictures affected the decision for seeking plastic surgery consultations and interventions		Total	*P* value
			No	Yes		
Surgeon advertisement affected the decision for seeking plastic surgery consultations and interventions	No	N	1023	417	1440	<.001
		%	84.1%	40.4%	64.1%	
	Yes	N	437	371	808	
		%	15.9%	59.6%	35.9%	
Employment status	Unemployed	N	270	211	481	.602
		%	22.2%	20.4%	21.4%	
	Student	N	413	342	755	
		%	34.0%	33.1%	33.6%	
	Self-employed	N	49	47	96	
		%	4.0%	4.6%	4.3%	
	Employed	N	484	432	916	
		%	39.8%	41.9%	40.7%	
Age (years)	12-20	N	165	115	280	.005
		%	13.6%	11.1%	12.5%	
	21-30	N	549	490	1039	
		%	45.1%	47.5%	46.2%	
	31-40	N	212	197	409	
		%	17.4%	19.1%	18.2%	
	41-50	N	184	176	360	
		%	15.1%	17.1%	16.0%	
	≥51	N	106	54	160	
		%	8.7%	5.2%	7.1%	

**Table 5.** Relationship Between Photograph Editing Applications in Seeking Consultation/Interventions and Surgeons

Surgeon advertisement affected the decision in seeking plastic surgery consultations and interventions		Photograph editing apps affected decision in seeking plastic surgery consultations and interventions		Total	*P* value
		No	Yes		
No	N	1190	250	1440	<.001
	%	81.2%	31.9%	64.1%	
Yes	N	275	533	808	
	%	18.8%	68.1%	35.9%	

**Table 6.** Relationship Between Educational Level of Participants and the Desire to Appear Better in Photos That Motivates Them to Undergo a Cosmetic Intervention

Educational level		Desire to appear better in photos motivates you to undergo a cosmetic intervention		Total	*P* value
		No	Yes		
No education	N	3	3	6	.872
	%	0.3%	0.3%	0.3%	
School (Primary, Secondary, high)	N	253	173	426	
	%	19.1%	18.7%	19.0%	
University (Bachelor, Master, PhD)	N	1066	750	1816	
	%	80.6%	81.0%	80.8%	

**Table 7.** Type of Social Media Used by the Participants

Value	Facebook (Menlo Park, CA)	Instagram (Menlo Park, CA)	Snapchat (Santa Monica, CA)	TikTok (Culver City, CA)	Twitter (San Francisco, CA)	Others	Total
N	50	422	855	242	455	224	2248
%	2.2	18.8	38.0	10.8	20.2	10	100.0

**Table 8.** Frequency of Social Media Usage and Its Relationship With Time and Other Characteristics

Patient information		Statistics	Never	Once	Multiple times	Every time	Total	*P* value
Educational level	No education	N	2	1	1	2	6	.659
		%	0.40%	0.20%	0.20%	0.40%	0.3%	
	School (Primary, Secondary, high)	N	109	94	144	79	426	
		%	18.20%	21.00%	18.10%	19.60%	19.0%	
	University (Bachelor, Master, PhD)	N	487	353	653	323	1816	
		%	81.40%	78.80%	80.70%	80.00%	80.8%	
Cosmetic contents to “look for” or “wanted to see” on social media	Before-and-after photographs	N	129	138	264	150	681	<.001
		%	21.57%	30.80%	33.08%	37.13%	30.29%	
	Information about procedures	N	107	90	134	80	411	
		%	17.89%	20.09%	16.79%	19.80%	18.28%	
	Videos about procedure	N	47	46	62	29	184	
		%	7.86%	10.27%	7.77%	7.18%	8.19%	
	Patient testimonials	N	49	40	59	36	184	
		%	8.19%	8.93%	7.39%	8.91%	8.19%	
	Information about surgeon’s	N	72	49	88	36	245	
		%	12.04%	10.94%	11.03%	8.91%	10.90%	
	Others	N	194	85	191	73	543	
		%	32.44%	18.97%	23.93%	18.07%	24.15%	

## DISCUSSION

The total number of participants in this current study was 2248. They reported that SM influenced them to seek cosmetic treatment and were comparatively more interested in doing cosmetic treatment, which showed a statistically significant relationship (*P* < .001).

It was reported that 56.7% (*n* = 1275) were not interested in doing surgical or non-surgical cosmetic interventions, while 43.3% were interested. For those influenced by the SM platforms, who were either interested or not interested in doing cosmetic interventions, Snapchat was the most influential SM platform, followed by Instagram. For those who were interested in doing cosmetic interventions, Snapchat represented (34.3%), Instagram (25.2%), TikTok (4.6%), Twitter (2.1%), Facebook (0.9%), and others (2.1%), while SM did not influence (30.1%) of them. And for those who were not interested in doing cosmetic interventions, Snapchat represented (11.2%), Instagram (6.0%), Twitter (2.1%), TikTok (1.8%), Facebook (0.4%), and others (1.3%), while no SM influenced 77.1%. This is in line with the different cross-sectional studies conducted by Suwayyid et al^6^ in 2020 among the Saudi population in different regions of Saudi Arabia, which showed that out of 911 participants, 38.6% intended to undergo plastic surgery, whereas 61.4% did not.

A similar result was found in a study conducted by Arab et al^16^ in 2018, with a sample size of 816 university Saudi female students; Snapchat had the highest influence on the decision to undergo a cosmetic procedure among the SM platforms.

The opposite was seen in 2019 by Al Ghoneim et al;^1^ for females visiting the facial plastic clinic at King Abdulaziz Medical City in Riyadh, Saudi Arabia, Instagram was the most influential SM platform (55%), followed by Snapchat (40%). SM is being used by people of different ages and different levels of education. Addressing the effect of SM platforms on young participants, greater than 12 years of age, was sought in this study to see how SM influenced them to consider having any future cosmetic intervention. We recognize that this age group in our community is active on SM and influencing others.

In a general and broader view, another study was done by Montemurro et al^17^ in 2015 on 500 patients to determine the impact of SM platforms on the everyday aesthetic plastic surgery practice, which showed almost all participants (95%) used the internet to gather information before consultation, and 68% admitted that SM is not only one source of information, but the first search method.

The rising influence of Snapchat may be due to the increasing number of plastic surgeons using Snapchat, along with the nature of Snapchat being a picture-oriented, rather than text-oriented, platform. However, further studies on the most common SM used by plastic surgeons in Saudi Arabia are needed.

Participants who reported that SM influenced them to undergo surgical or non-surgical cosmetic interventions were comparatively more interested in doing so, which showed a statistically significant relationship (*P* < .001). On one hand, 6% (*n* = 135) of respondents underwent surgical cosmetic interventions such as liposuction, breast augmentation, and rhinoplasty and 94% (*n* = 2113) have not undergone any surgical cosmetic intervention. For both, Snapchat was the most influential SM, followed by Instagram. On the other hand, 15.2% (*n* = 342) of responders underwent non-surgical cosmetic interventions such as botulinum toxin and fillers, and 84.8% (*n* = 1906) have not undergone any non-surgical cosmetic interventions. For both, Snapchat was the most influential SM platform, followed by Instagram.

A similar result was seen in Suwayyid et al;^6^ the majority, 87%, had no history of plastic surgery, and 13% had an account with plastic surgery. In this current study, it was reported that 35.9% (*n* = 808) of the participants confirmed that surgeons’ advertisements affected their decision in seeking plastic surgery consultations and treatment, which was higher in participants who reported that surgeons’ advertisements on SM platforms influenced them to seek cosmetic treatment than those who said that it did not influence them, which was statistically significant (*P* value <.001). This goes in line with a study by Suwayyid et al^6^ in 2020 regarding the factors affecting a participant's decision for undergoing plastic surgery, whether surgical or otherwise, in Saudi Arabia. This study showed that the before-and-after pictures on SM were the most motivating factor to undergo cosmetic procedures, representing 44.1%. On the other hand, a study conducted by Arab et al^16^ showed that advertisements influenced 48.5% of respondents on SM to undergo cosmetic treatments, which led us to consider SM, especially Snapchat and Instagram, as having a critical role in influencing users who are interested in undergoing surgical or non-surgical cosmetic interventions (87.9%). Reaching plastic surgeons and asking questions has become easier with the existence of SM, and this was most influenced by surgeons’ reputations and SM advertisements.

These results reflect Basa et al (2021).^18^ They also found that a list of the top 33 facial plastic surgery Instagram profiles and SM tracking tools (Awario, Belarus) was compiled based on the number of followers and “reach” obtained on September 22, 2019. The top later categorized 18 posts into clinical or professional, lifestyle, and patients at the time of data extraction. The average number of “likes,” as a percentage of total followers, was calculated for each category for each profile. Clinical pictures and patient posts were 42.78% and 41.94%, respectively. Way of life substance was 19.05%. Followers interacted with clinical posts the foremost, having an average of 6.79% of follower interaction. Patient and lifestyle posts had 2.88% and 3.81%, respectively.

SM creates a good rapport between patients and plastic surgeons by sharing clinical information with their experiences. Of the participants, 35.05% (n=788) reported that before-and-after pictures affected their decision to seek plastic surgery consultations and interventions, and that a surgeon's advertisement was an influential factor (59.6%) (*P* < .001).

In addition, a study conducted among adult Saudi females and males who underwent or have the desire to undergo a cosmetic procedure with a total of 911 participants showed that before-and-after pictures on SM and advertisements of plastic surgeons themselves influence a participant's desire for cosmetic intervention, and was significantly associated with undergoing plastic surgery.^6^

These results reflect those of other studies that were conducted among patients attending cosmetic clinics at King Abdulaziz University Hospital in Riyadh, Saudi Arabia, with 399 participants (65.7%) reporting that before-and-after photos on SM had affected their decision to undergo plastic surgery.^5^

There is evidence that 34.8% believe photograph-editing applications affected their decision to seek plastic surgery consultation and interventions, which goes in agreement with the study by Aldosari et al.^5^ The survey asked socio-demographic information and the reason for plastic surgeries. Three hundred ninety-nine patients took an interest in the study. Of all participants, 60.4% agreed on the effect of the surgeons’ self-advertisement on the trend of plastic surgeries; 53.4% answered yes to cosmetics television programs influencing the trend of plastic surgeries; 65.7% of the participants said yes to before-and-after photos of SM affecting the direction of cosmetic surgery, and 54.1% of the participants said yes to needing to look superior in selfies as a reason for the rise of cosmetic surgery.

In addition, a study conducted to evaluate face-editing applications, motivations in usage and influences toward pursuing cosmetic procedures, and attitude toward applications reported that 32.9% admitted to using face-editing applications. Similar to our results, this study showed that most participants confirmed that these applications played a role in pursuing cosmetic procedures (56.5%).^19^

Our study showed that there is a significant association between the participants who used applications that have photograph-editing abilities and filters multiple times, and those who were influenced to undergo cosmetic surgery. This was similar to the study published in 2019 by Chen et al,^3^ which proved that there is a strong positive correlation between the number of hours spent on these applications and an increased desire to undergo cosmetic surgery. The majority have been using SM for more than 5 hours per day, and this is consistent and compatible with our findings (44.4%).

Also, there was a study conducted by Gould et al^20^ that mentioned that people use SM approximately 2 hours per day for many purposes, including increasing knowledge about procedures and conditions regarding plastic surgery. The analysis of time spent on SM showed that about 35.5% of the participants used SM multiple times, whereas 26.6% never used it. The most commonly looked up or searched cosmetic content was “before-and-after photographs of cosmetic treatment” (30.29%) followed by information about procedures (18.28%).

A study conducted in a single aesthetic practice of 2 plastic surgeons by surveying 100 consecutive patients showed that the top 3 preferred contents posted on the practice’s SM feed, among 80% of patients, are contests to win a free treatment or product (31% very interested; 49% interested), before-and-after photographs (24% very interested; 56% interested), and information about the practice (13% very interested; 68% interested).^8^

Another 2-cohort study was conducted in the aesthetic plastic surgery clinic and public department of plastic surgery by surveying 313 patients and questioning them about information searched by patients on the internet. It was reported that the patients were frequently seeking information about the clinic and reviews of other patients,before-and-after photographs, and looking for information about the surgeon’s qualifications, particularly, specialty, training, courses, congresses, etc.^13^

Forty-six percent think that the photograph- editing application made them look better and more confident to post/share their photos on SM. In agreement with our study, Chen et al^3^ reported that using specific applications such as YouTube, Tinder (Los Angeles, CA), and Snapchat photograph filters increased the acceptance of cosmetic surgery. The use of other applications, including WhatsApp (Menlo Park, CA) and Adobe Photoshop (San Jose, CA) was associated with significantly lower self-esteem scores. Those findings indicate that increased investment in SM platforms was associated with increased consideration of cosmetic surgery. The use of certain SM and editing applications may be associated with an increased acceptance of cosmetic surgery.^3^ Technology is a rapidly evolving field, and many SM platforms are always developing.

This study is conducted to evaluate the effect of different SM platforms. The strength of our study is that we studied the recent SM platforms such as TikTok. Patients’ attitudes toward these platforms, and whether they are really affected by them or not, will help to increase the population's awareness of different types of cosmetic procedures and will also help in improving patient-surgeon relationships. This study is limited to the evaluation of impact among the general population and not plastic surgeons; so further studies to evaluate the impact of SM platforms among plastic surgeons are encouraged.

Many SM platforms were not included in our study, as they are less common. Also, social media is a rapidly growing field and there will be many new platforms that will be developed in the future. Hence, future works should elucidate whether patient attitude changes over time as new platforms are created.

## CONCLUSIONS

This study aimed to evaluate the effect of different SM platforms on plastic cosmetic surgery in Saudi Arabia. Our analysis showed that those influenced by SM platforms to seek cosmetic treatment were comparatively more interested in cosmetic treatment than those not influenced by SM, with Snapchat being the most influential platform. Therefore, further studies to evaluate the impact of SM platforms among plastic surgeons are encouraged.

## Supplementary Material

ojad002_Supplementary_Data
