# Supplementary material for: The Effect and Implication of Social Media Platforms on Plastic Cosmetic Surgery: A Cross-sectional Study in Saudi Arabia From 2021 to 2022
Source: Aesthet Surg J Open Forum. 2023 Jan 18;5:ojad002. doi: 10.1093/asjof/ojad002 (PMC10045880; doi:10.1093/asjof/ojad002)
Supplement: ojad002_Supplementary_Data [file ojad002_supplementary_data.pdf]

Annex

We are group of doctors conducting a research to evaluate the effect of social media platforms on plastic cosmetic surgery in Saudi Arabia, we ask you ladies and gentlemen, to answer the questions of our survey to help us to complete our research

(Thank you for your precious time)

(Questionnaire by English language)

1-Age:
O 12-20
O 21-30
O 31-40
O 41-50
O 51+

2- Gender:
O Male
O Female

3-Nationality:
O Saudi
O Non-Saudi

4-Regions:
O North
O East
O West
O South
O Center

5-Marital status:
O Married
O Single
O Divorced
O Widow

6-Education level:
O No education
O School (Primary, Secondary, high)
O University (Bachelor, Master, PHD)

7-Employment status:

O Unemployed
O Employed
O Self-Employed
O Student

8-Monthly income:
O I don’t have income
O <5000
O 5000-10.000
O 11.000-20.000.
O 21.000-40.000
O >40.000

9- How often do you use social media per day?
O Less than 2 hours.
O 2-5 hours.
O More than 5 hours

10-Which of the following social media platforms do you use the most?
O Instagram
O Twitter
O Snapchat
O TikTok
O Facebook
O Others

11- Do you follow any plastic surgeons on any social media platforms?
O Yes
O No

12-Are you interested in doing a cosmetic treatment either surgical or non surgical?
O Yes
O No

13-Have you ever had a surgical cosmetic intervention such as (liposuction, breast augmentation, rhinoplasty, etc.)?
O Yes
O No

14-Have you ever had a non-surgical cosmetic intervention such as (botulinum toxin, fillers, etc)
O Yes
O No

15- Which of the following social media platforms influenced you to consider doing surgical or non-surgical cosmetic interventions?
O Instagram

O Twitter.
O Snapchat.
O TikTok.
O Facebook.
O Others
O I was not influenced by any social media platforms

16-Reaching surgeons and asking questions has become easier with the existence of social media?
O Yes
O No

17-What makes you accept visiting a plastic clinic for a consultation?
O Someone's recommendation
O Surgeon reputation
O Social media advertisement
O Self decision
O Photograph editing applications
O others

18-Has your salary affected your decision to undergo plastic cosmetic interventions?
O Yes
O No

19-Surgeon's advertisement affected your decision in seeking plastic surgery consultations and interventions?
O Yes
O No.

20-Before-and-after pictures affected your decision in seeking plastic surgery consultations and interventions?
O Yes
O No.

21-Photograph editing applications affected your decision in seeking plastic surgery consultations and interventions?
O Yes
O No.

22-Photograph editing applications made you look better and more confident to post/share it in different social medias?
O Yes
O No.

23-The desire to appear better in pictures and selfies encourages you to undergo a cosmetic intervention?

O Yes
O No.

24-The area of interest for cosmetic intervention:
O Face.
O Breast.
O Abdomen.
O Arms.
O Thighs
O other

25-Most important cosmetic contents that you look for or you want to see on social media platforms?
O Before and after photographs
O Information about procedures.
O Videos about procedure.
O Patients testimonials.
O Information about surgeon's
O other

26-If you use any applications that have the photograph editing abilities and filters, how frequently do you apply them?
O Never.
O once.
O multiple times.
O everytime

Questionnaire by Arabic language
(الاستبيان باللغة العربية)

نحن مجموعة من الأطباء لدينا دراسة بحثية متعلقة بتأثير منصات وسائل التواصل الاجتماعي على العمليات والإجراءات التجميلية في المملكة العربية السعودية، نطلب منكم تكرمًا المشاركة في الإجابة عن هذه الأسئلة لمساعدتنا في البحث.
(وشكرًا لوقتكم الثمين)

البيانات الشخصية:
١) العمر :
- 12-20
- 21-30
- 31-40
- 41-50
- أعلى من 51

٢) الجنس
- ذكر
- انثى

٣) الجنسية :
- سعودي/ة
- غير سعودي/ة

٤) المنطقة السكنية:
- المنطقة الشمالية
- المنطقة الشرقية
- المنطقة الغربية
- المنطقة الجنوبية
- المنطقة الوسطى

٥) الحالة الاجتماعية:
- أعزب
- متزوج/ة
- مطلق/ة
- أرمل/ة

٦) مستوى التعليم:
- غير متعلم/ة
- دراسي (ابتدائي، متوسط، ثانوي)
- جامعي (بكالوريوس، ماجستير، دكتوراة)

٧) الحالة الوظيفية:
- غير موظف/ة
- موظف/ة
- أعمال حرة
- طالب/ة

٨) الدخل الشهري:

- ليس لدي دخل
- اقل من 5000
- من 5000 - 10.000
- من 11.000 - 20.000
- من 21.000 - 40.000
- أكثر من 40.000

٩) كم مرة تستخدم شبكات التواصل الاجتماعي في اليوم الواحد؟

- أقل من ساعتين
- من ساعتين لخمسة ساعات
- أكثر من خمسة ساعات

١٠) أي من شبكات التواصل الاجتماعي التالية تستخدمها أكثر من غيرها؟

- انستقرام Instagram
- تويتر Twitter
- سناب شات Snapchat
- تيك توك TikTok
- فيس بوك Facebook
- أخرى

١١) هل تقوم بمتابعة أي جرّاح تجميلي على أي من شبكات التواصل الاجتماعي؟

- نعم
- لا

١٢) هل لديك الرغبة في إجراء أي علاج تجميلي سواء بتدخل جراحي أم غير جراحي؟

- نعم
- لا

١٣) هل سبق لك أن خضعت لأي تدخل جراحي تجميلي مثل (شفط الدهون ، تكبير أو تصغير الثدي، تجميل الأنف ، إلخ)؟

- نعم
- لا

١٤) هل سبق لك أن خضعت لتدخل تجميلي غير جراحي مثل (البوتوكس، الفيلر، إلخ)؟

- نعم
- لا

١٥) أي من وسائل التواصل الاجتماعي التالية كان لها التأثير في جعلك تأخذ بعين الاعتبار القيام بتدخل تجميلي جراحي أو غير جراحي؟

- انستقرام Instagram
- تويتر Twitter
- سناب شات Snapchat
- تيك توك TikTok
- فيس بوك Facebook
- وسائل تواصل أخرى
- لم أتأثّر بأيّ من وسائل التواصل الاجتماعي

١٦) هل أصبح الوصول إلى الجراحين وطرح الأسئلة أسهل مع وجود وسائل التواصل الاجتماعي؟
- نعم
- لا

١٧) ما الذي يجعلك تُقبل على زيارة عيادات التجميل للحصول على استشارة؟
- توصية شخص ما
- سُمعة الجرّاح
- إعلان على وسائل التواصل الاجتماعي
- قرار ذاتي
- تطبيقات تحرير أو تعديل الصور
- غير ذلك

١٨) هل يؤثر دخلك على قرارك بالخضوع لتدخلات تجميلية؟
- نعم
- لا

١٩) هل أثّرت إعلانات الجراحين على قرارك في طلب الاستشارات والتدخلات التجميلية؟
- نعم
- لا

٢٠) هل أثّرت رؤيتك للصور (قبل وبعد) التدخلات التجميليّة، على قرارك في طلب الاستشارات والتدخلات التجميلية؟
- نعم
- لا

٢١) هل أثرت تطبيقات تعديل الصور على قرارك في طلب الاستشارات والتدخلات التجميلية؟
- نعم
- لا

٢٢) هل جعلتك تطبيقات تعديل الصور تبدو أفضل وأكثر ثقة في نشر / مشاركة الصور في شبكات التواصل الاجتماعية المختلفة؟
- نعم
- لا

٢٣) الرغبة في الظهور بشكل أفضل في الصور والسيلفي تشجعك على الخضوع لتدخل تجميلي؟
- نعم
- لا

٢٤) المنطقة التي تود إجراء تدخل تجميلي لها؟
- الوجه
- الثدي
- البطن
- الذراعين
- الفخذين
- أخرى

٢٥) أهم المعلومات التي تبحث عنها أو تودّ رؤيتها في تطبيقات التواصل الاجتماعي فيما يخص التدخلات التجميلية ؟

- صور قبل وبعد الإجراء التجميلي
- معلومات عن الإجراء أو العملية التجميلية
- مقاطع فيديو عن الإجراء التجميلي
- توصية أحد المرضى
- معلومات عن الجراحين
- أخرى

٢٦) إذا كنت تستخدم أي تطبيقات تحتوي على فلاتر لتعديل الصور فكم مرة تستخدمها؟

- لم أستخدمها أبدًا
- مرة واحدة
- عدة مرات
- دائمًا
